# Variation in Price of Cardiovascular and Diabetes Medicine in Indonesia, and Relationship with Quality: A Mixed Methods Study in East Java

**DOI:** 10.4269/ajtmh.22-0692

**Published:** 2023-05-09

**Authors:** Elizabeth Pisani, Aksari Dewi, Anna Palagyi, Devarsetty Praveen, Bachtiar Rifai Pratita Ihsan, Ayuk Lawuningtyas Hariadini, Diana Lyrawati, Sujarwoto, Asri Maharani, Gindo Tampubolon, Anushka Patel

**Affiliations:** ^1^ The George Institute for Global Health, University of New South Wales, Sydney, Australia;; ^2^ The George Institute for Global Health, Hyderabad, India;; ^3^ Department of Pharmacy, Faculty of Medicine, Brawijaya University, Malang, Indonesia;; ^4^ Department of Public Administration, Brawijaya University, Malang, Indonesia;; ^5^ Faculty of Biology, Medicine and Health, University of Manchester, Manchester, United Kingdom;; ^6^ Global Development Institute, University of Manchester, Manchester, United Kingdom

## Abstract

Lower-middle income Indonesia, the world’s fourth most populous country, has struggled to contain costs in its mandatory, single-payer public health insurance system since the system’s inception in 2014. Public procurement policies radically reduced prices of most medicines in public facilities and the wider market. However, professional associations and the press have questioned the quality of these low-cost, unbranded generic medicines. We collected 204 samples of four cardiovascular and one antidiabetic medicines from health facilities and retail outlets in East Java. We collected amlodipine, captopril, furosemide, simvastatin, and glibenclamide, sampling to reflect patients’ likelihood of exposure to specific brands and outlets. We recorded sales prices and maximum retail prices and tested medicines for dissolution and percentage of labeled content using high-performance liquid chromatography. We conducted in-depth interviews with supply chain actors. All samples, including those provided free in public facilities, met quality specifications. Most manufacturers make both branded and unbranded medicines. Retail prices varied widely. The median ratio of price to the lowest price for an equivalent product was 5.1, and a few brands sold for over 100 times the minimum price. Prices also varied between outlets for identical products because retail pharmacies set prices to maximize profit. Because very-low-cost medicines were universally available and of good quality, we believe richer patients who chose to buy branded products effectively protected medicine quality for poorer patients in Indonesia because manufacturers cross-subsidize between branded and unbranded versions of the same medicine.

## INTRODUCTION

As middle-income countries expand their efforts to provide Universal Health Coverage, pressure on government health budgets has increased.^1^ Cost-containment measures often include procurement policies that seek to bring down the price of medicines in the public system, including by increasing the proportion of unbranded generic medicines used.^2^ However, sharp falls in prices have, in some cases, led to questions being raised about the quality of publicly procured medicines.^3^

Previous studies indicate that physicians and patients continue to question the quality of unbranded generic and other low-cost medicines, despite substantial and growing evidence that they are therapeutically equivalent or superior to originator brands (i.e., the brand that originally held the patent for this medicine and formulation) and other branded products.^4^^–^^6^ Global reviews show that this is especially likely to be the case in low- and middle-income countries, where medicines provided for free in public health systems are particularly mistrusted.^7^^–^^9^

In Indonesia, the world’s fourth most populous nation, a national medicines procurement system was introduced in 2014 in support of a new, mandatory, single-payer health insurance system that, by mid-2022, covered over 80% of the population.^10^ The single-winner auction system, known as e-catalog, created intense competition among domestic producers of unbranded generic medicines and drove prices for common medicines for chronic diseases sharply lower. Nearly 80% of the medicine procured in 2017 through e-catalog had fallen in price compared with 2013; prices of 39% of these medicines fell by more than 50%.^11^ The downward trend has continued in more recent years. For example, the price of the blood pressure–lowering medicine amlodipine fell from 440 rupiah per 10-mg tablet in 2013 to 70 rupiah by 2022 (US$ 0.045 to 0.005); the cholesterol control medicine simvastatin fell from 180 rupiah per 10-mg tablet to 68 rupiah (US$ 0.018 to 0.005) over the same period.^12^ Falling prices have led one multinational generics producer to withdraw from the Indonesian market entirely.^13^ The domestic industry association has warned that unsustainably low prices may threaten the quality and sustainability of supply, and in the early years of the new procurement system, newspapers and consumer associations regularly called into question the quality of medicines provided in public clinics and hospitals.^14^^–^^17^

Prices for many unbranded generics (also referred to as International Non-proprietary Name [INN] generics) in the private market fell in tandem with public procurement prices. However, many domestic pharmaceutical companies that hold market authorizations to sell these unbranded products also sell branded versions of the same medicines. Under Indonesian regulations these should be formulated identically with the unbranded product registered to the same market authorization holder but command many times the price in the market.

The procurement and flow of medicines from producer to patient for those using Indonesia’s public health insurance system at the time of the study are described in detail elsewhere.^18^ These medicines are provided free to patients at point of care. Patients who are not insured, or who do not want to queue at public facilities for free medicines, can pay for medicines at retail pharmacies. Patients may buy more expensive medicines because they are prescribed by a doctor or suggested by a health care worker or pharmacist who may be rewarded with increased profits or through pharmaceutical company incentives if the patient takes a more expensive medicine.^19^ They may also choose more expensive medicines, even when a cheaper version is offered, because they associate higher prices with better quality.^13^

The maximum retail price must be printed on the primary packaging of all medicines in Indonesia. Pharmaceutical companies are free to set the maximum retail price for branded medicines for which they hold market authorizations at any price of their choosing. Regulations passed in 2015 cap the maximum retail price for unbranded generics at the public procurement price plus 28%.^20^ In practice, prices charged to patients are not always in line with the maximum retail price; products are sold both above and below that price.

There has, to our knowledge, been only limited description of price variation in the Indonesian medicine market^21^ and no independent investigation of the association between medicine price and medicine quality in Indonesia. In this study, we investigate the relationship between price and quality for 204 samples of five medicines for cardiovascular disease and diabetes in Malang district, East Java, Indonesia. We further describe the variation in medicine prices by branded status, brand identity, and point of dispensing to patients.

## MATERIALS AND METHODS

The study centered on eight villages in Malang district, a semi-rural district in Indonesia’s second most populous province, which were selected because they were the site of a household census of the prevalence of risk for cardiovascular disease.^22^ The study methods are reported in detail elsewhere, according to MEDQUARG guidelines.^23^ The MEDQUARG checklist is available in the study archive.^24^

Briefly, we designed an exposure-based sample frame. We selected the five medicines that patients at high risk for cardiovascular disease most commonly reported consuming in an earlier household census.^22^^,^^25^ These were the cardiovascular medicines amlodipine, captopril, furosemide, and simvastatin as well as the anti-diabetes medicine glibenclamide. (For brevity, we refer to these as “study medicines” throughout the paper.) We then triangulated data from the patient survey with data from pharmacies, the public procurement system, and pharmaceutical marketing tracking systems and constructed a sample frame based on the estimated likelihood that patients at high risk for cardiovascular disease and diabetes in the study area would consume a particular medicine from a particular source. Details of sample frame construction can be found in the study archive, File 02.^24^

### Medicine outlets.

We collected samples from the district medicine warehouse (1/1), which supplies all public primary health care clinics (known as *pusat kesehatan masyarakat* or *puskesmas*) and their outreach services, the district hospital (1/1), private doctors and midwives (8/30 of those reporting selling study medicines), private pharmacies (55/75 pharmacies), and over-the-counter (OTC) medicine shops (2/3 of those found to sell study medicines). In addition, we collected any medicines provided for free to patients in *puskesmas* that were procured directly from distributors or pharmacies using capitation funds (2/2).

We note that all of the study medicines are regulated as prescription-only. This means that private pharmacies are only allowed to sell them to patients with prescriptions. Private health care providers and OTC medicine shops are not technically allowed to sell them in the study area. However, they commonly do so and were therefore included in the sample. For more details see Dewi et al.^23^

### Sample collection strategy.

All samples were collected from February to May 2021. We sampled overtly from public facilities, taking one sample of every available brand of study medicine. In pharmacies and OTC medicine shops, we used mystery shoppers posing as patients or family or friends of patients. At each outlet, shoppers requested a single medicine or a combination consistent with common clinical needs. To approximate the market distribution of medicine price points, they signaled their desire for cheaper or more expensive medicines using phrases such as “Minta yang terjangkau” (I want something affordable) or “Ada yang paten?” (Do you have anything “patent”? – the term commonly used in Indonesia to signify a branded product).

If the sample frame called for clinically incompatible combinations or repetitions (for example cheap and expensive versions of the same product) from a single outlet, different mystery shoppers were used.

### Sample handling and testing.

All the study medicines are normally packaged in strips/blisters of 10 tablets. We aimed to collect 40 tablets per sample but accepted a minimum of 30 tablets.

On exiting the outlet, collectors put each sample in a sealable plastic bag marked with a pre-printed barcode. The barcode was scanned, and field-related data were entered into a form pre-loaded onto the shoppers’ mobile phones using open-source KoboCollect software.^26^^,^^27^ Further data entry, including product photographs and details of market authorization holder, manufacturer, registration number, and expiry date, took place at the end of the day using a second form linked by the same barcode. The ODK-format data collection forms are available in the study archive, Files 03 and 04.^24^

Research team members inspected packaging visually. No reference packaging was available for comparison, so visual inspection, using a magnifying glass as necessary, was limited to checking for anomalies such as misspellings and discrepancies in formatting of batch numbers and expiry dates.

Samples were stored in a temperature-controlled environment for an average of 21 days, batched, and sent (with a temperature logger) for testing to PT Equilab International, an ISO/IEC 17025-certified private laboratory in Jakarta. Samples were tested using USP 42 NF 37 monographs and USP reference standards. Methods were validated for all active pharmaceutical ingredients (APIs) before testing. The full protocols for each molecule are available in the study archive, Files 09–14.^24^

Laboratory staff inspected tablets visually, noting shape, color, lettering, and other defining characteristics. Chemical analysis was performed for determination of identity, assay (% of labeled active ingredient), and dissolution (% of labeled active ingredient in the tablet dissolved over time). For all APIs, assay testing was by ultraviolet high-performance liquid chromatography (Aliance 2695 with UV Detector 2489 for amlodipine, glibenclamide, furosemide, and simvastatin; Aliance 2695 with Photodiode Array Detector 2996 for captopril; Waters). Dissolution was by ultraviolet-visible spectrometry (Shimadzu UV-1800) with the exception of glibenclamide, for which dissolution was tested by high-performance liquid chromatography (Aliance 2695 with UV Detector 2489; Waters).

Assay testing was duplicated; the reported result is the average of the two tests. We could not afford to test for uniformity or impurities.

Staff conducting the tests differed from those handling the packaged product but could see any defining marks on tablets or capsules. Testing took place April to August 2021, an average of 95 days after sample collection.

Results from the certificate of analysis were entered into a database by study staff using the sample barcode as identifier. Raw dissolution data were added to the database at a later date, delaying stage 2 dissolution. Where necessary, this was undertaken in March 2022.

Table 1 shows the definitions used for compliance with specifications, following USP 42 NF 37 limits.

**Table 1:** Limits of compliance, United States Pharmacopeia 42 (% of declared content)

API	Assay (%)	Dissolution [Q] (%)	Stage 1 dissolution [Q+5] (%)
Amlodipine	90–110	75	80
Captopril	90–110	80	85
Furosemide	90–110	80	85
Glibenclamide	90–110	70	75
Simvastatin	90–110	75	80

API = active pharmaceutical ingredient.

If any one of six pills included in stage 1 dissolution fell below the stage 1 threshold of Q + 5, we continued to stage 2 testing using additional six tablets. The sample was considered out of specification if 1) the assay fell outside the stated limits, OR 2) any single tablet fell below the Q threshold −25 in dissolution testing, OR 3) any three tablets fell below Q threshold −15 in dissolution testing, OR 4) the average of 12 tablets fell below the Q threshold in stage 2 dissolution testing.

### Panel pricing data.

With the written consent of pharmacy owners, we collected monthly data from two private pharmacies on all brands of stocked study medicines between March and October 2021. One was a local branch of a national chain; the other was an independent pharmacy. From their stock management systems, pharmacists provided us with volumes received and volumes dispensed by brand as well as buying prices and selling prices.

We calculated the profits by brand by multiplying sales volumes by margin (selling price − buying price). We estimated the list price for each medicine by subtracting the maximum margin of 28% (10% tax and 18% “service fee”) allowed by Indonesian regulations^20^ from the maximum retail price and then estimated the percent discount at which each product was acquired by comparing the buying price with the list price.

### Quantitative data analysis, medicine quality, and pricing survey.

The field data form, the product data form, and the laboratory data were merged on barcode number using Stata 17.0 software (StataCorp LLC, College Station, TX). Stata 17.0 was also used for reproducible cleaning and coding and to generate descriptive statistics and graphs.

To be able to compare price variation between molecules sold at different prices, we calculated the ratio of each sample price to the lowest recorded price paid for the medicines. In most pricing analyses we excluded “zero” retail prices (i.e., medicines provided free through the public health system). In the case of the district hospital, which provides medicines free to insured patients but charges uninsured patients for the same medicines, we priced their medicines at the price paid by the uninsured.

In the analysis comparing price with quality, we included public sector samples at the price charged to uninsured patients for hospital samples and at the procurement price for samples from the district warehouse or bought by *puskesmas* with capitation funds.

In comparing maximum retail prices, we kept just one instance of each unique market authorization number (reflecting a single medicine, dosage, formulation, market authorization holder, and brand or unbranded generic; *N* = 83). In a few cases, because prices may be recalibrated over time, there was more than one maximum retail price per market authorization number. In these cases, we used the maximum retail price value of the sample with the longest time to expiry as a proxy for the most recent version of the product.

When calculating the difference between the sales price and the maximum retail price at the sample level, we used the price and maximum retail price for the individual sample.

### Indonesian market data.

We extracted data, including market authorization holder, manufacturer, branded status, and brand name (if applicable), for all versions of the study medicines registered for sale in Indonesia from the open-access database maintained by the Indonesian medicine regulator.^28^ With permission from Universitas Pancasila, we merged information on holding companies for market authorization holders from a database maintained by the Faculty of Pharmacy’s Center for Pharmaceutical Policy and Services Studies using Stata 17.0 software.

### In-depth interviews.

We conducted in-depth interviews between June 2020 and May 2021 with purposively selected individuals with knowledge of medicine management in public and private sectors (Supplemental Table 1 gives details). Potential participants were approached by E-mail, outlining details of the study and requesting their participation; those who agreed to participate provided verbal or written consent. The interviews, conducted in Indonesian, were recorded and transcribed verbatim. Because the in-depth interviews took place during the COVID-19 pandemic, most interviews were conducted remotely by telephone, WhatsApp, or using the Google Meet platform.

Topics covered included the influence of price and other incentives on choice of medicines and perceptions of quality.

### Study permissions.

The Malang District Department of Health gave written permission for the study (070/1102/35.07.103/2020). The study also received ethics approval from the Ethical Committee, Ministry of Research, Technology, and Higher Education, Medical Faculty of Brawijaya University (No. 83/EC/KEPK/04/2020) and the Human Research Ethics Committee of University of New South Wales, Sydney (HC200148). Patients were not directly involved in the design, conduct, or reporting of this study. The results were reported to the Indonesian medicine regulator within 1 month of completion of assay and stage 1 dissolution testing.

## RESULTS

In the results section, we integrate data from in-depth interviews where appropriate to provide additional context for quantitative analysis.

### Relationship between price and quality.

**Figure 1.**
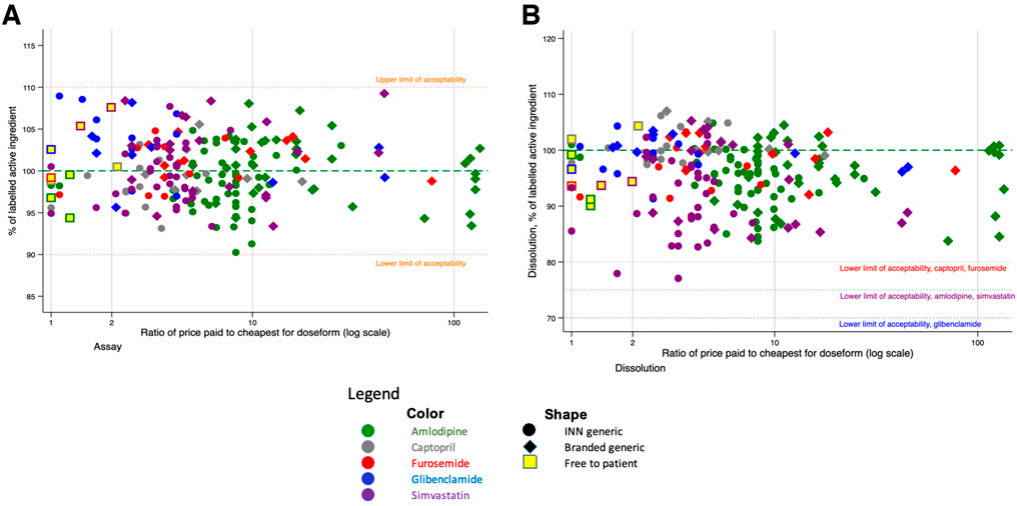
Relationship between price paid and assay (**A**) and dissolution (**B**) testing results, of 204 cardiovascular disease medicines samples by branded status.

Figure 1 plots the results of pharmacopeial tests against the relative price of each product to the cheapest for the medicine and dosage. The square markers indicate the medicines provided free to patients in the public health system; these are priced at the public procurement price. All samples met the quality specifications for both assay (Figure 1A) and dissolution (Figure 1B). Thus, there was no relationship between price and quality for any molecule in our study. (The online version of this and all other graphs use colors to differentiate between the different medicines.)

Interviewees were not consistent in their perception of the relationship between quality and price. One doctor, who provided services to poorer patients in the study area shortly before the introduction of the national health insurance program, was distrustful of low-priced medicines.

*If I thought a patient was doing ok with a medicine [priced] at 2000 or 3000 per strip [1.3–2 cents per pill], then I’d keep them on it. But in several cases, it just didn’t work. So that’s why I had more expensive medicines, branded medicines. So I’d say to the patient “Ma’am, this is a branded medicine, from [Company X] or [Company Y], it costs this much, do you want it or not?” And she’d say “Ya, OK doc, if it’s a good medicine.” And it would work. It worked when she used a branded medicine. So that’s why I don’t know if the contents of that 3,000 rupiah medicine is lower than the branded one, or what?*

Private doctor 1

A public-sector pharmacist noted that quality of unbranded products in the public procurement system appeared to have improved in recent years.

*Interviewer: What’s your opinion of the quality of medicines on e-catalog?*

*Respondent: It’s variable. I’ve been buying since the beginning, in 2014, and it was all over the place. Like, there was amox[icillin] I think, that each strip only had eight or nine pills, so a box wasn’t 100 [tablets]. It was only 80 or 95… There was also antacid that was too thick to pour, and [tablets] that were crushed up, all kinds of stuff. But now, it’s getting better each year, the quality is constantly improving.*

Public Sector Pharmacist

### Product variation in the Indonesian market.

All registered brands of all study medicines in Indonesia were manufactured domestically. There were 71 manufacturers making any study medicine at the time of data collection. Some of them produced medicines under contract for several market authorization holders. A total of 80 market authorization holders (grouped into 70 holding companies) registered at least one study medicine. Altogether, at the time of the study, there were 110 branded and unbranded versions of amlodipine registered in Indonesia, 56 of simvastatin, 17 of captopril, 13 of furosemide, and 12 of glibenclamide.

Because many holding companies sold more than one study medicine, we found a total of 133 holding company–molecule pairs. For example, Holding Company A–amlodipine constitutes one pair; if Holding Company A also makes simvastatin, this would constitute a second pair.

**Figure 2.**
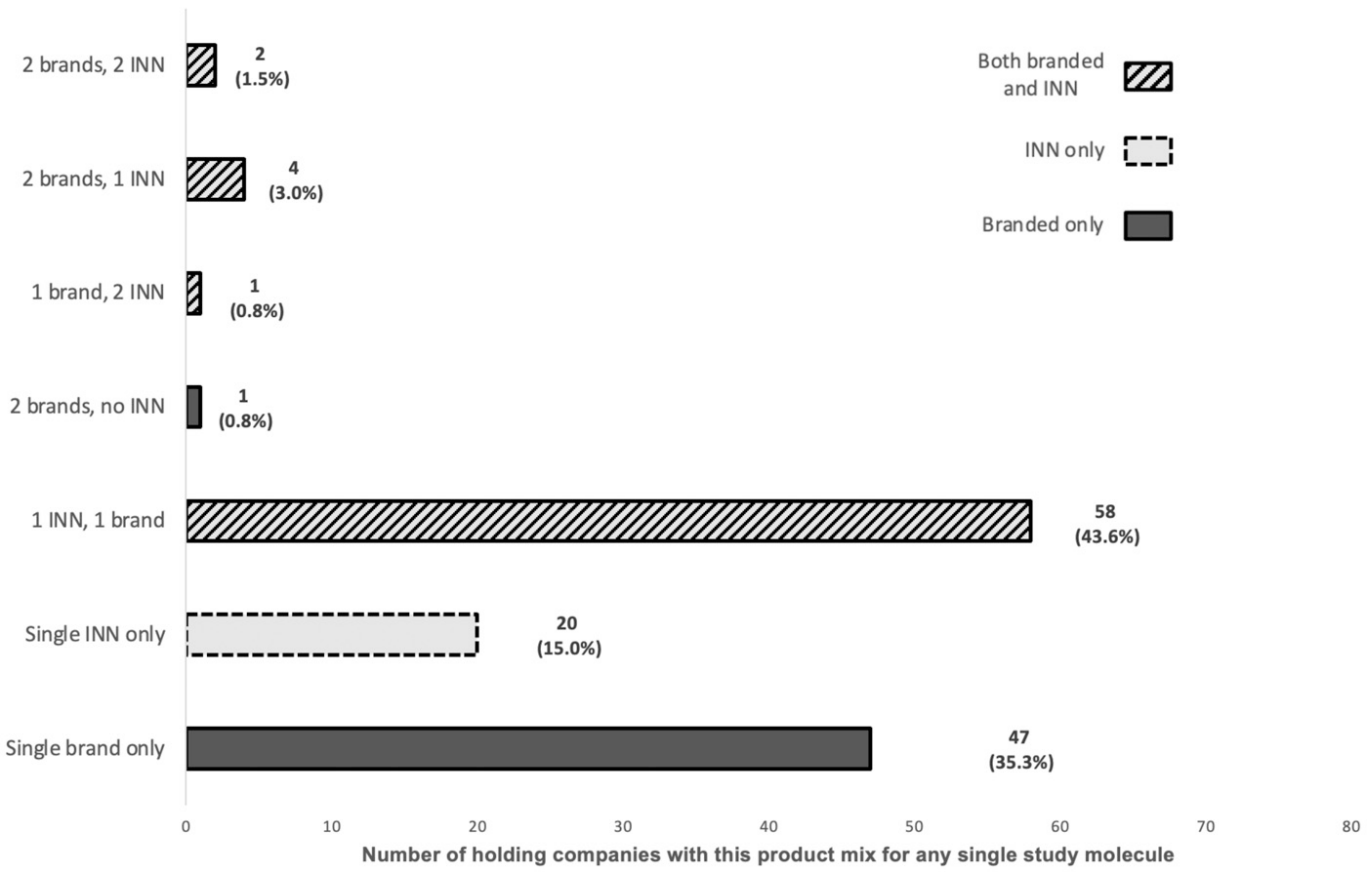
Distribution of product mixes registered by holding company for any individual study molecule.

Looking at holding company–molecule pairs in the Indonesian market, we found that just over half were singletons; either a single brand (35.3%) or a single unbranded (INN) product (15%) (Figure 2). For most of the rest (43.6%), the holding company had registered one branded and one INN version. The remainder registered two brands or three or four brands/INN versions of the same molecule. In a few cases a single holding company registered a medicine to two or three different market authorization holders, allowing them to make more than one INN version of the same medicine.

### Sample distribution in the medicine pricing and quality survey.

Table 2 shows the number of samples and brands by medicine and type of source. In accordance with the sampling strategy reflecting population exposure, unbranded generics outnumbered branded generics in both number of samples and variety of products.

**Table 2:** Details of products collected in study

API	Samples			Brands			Unique products (brand and dose)		
	Branded	INN	Total	Branded	INN	Total	Branded	INN	Total
Amlodipine	35	53	88	14	16	30	18	22	40
Captopril	8	14	22	1	4	5	1	6	7
Furosemide	11	10	21	4	4	8	4	4	8
Glibenclamide	9	12	21	4	4	8	4	2	6
Simvastatin	19	33	52	6	11	17	7	15	22
Total	82	122	204	29	39	68	36	49	83

API = active pharmaceutical ingredient; INN = International Non-proprietary Name.

### Variation in maximum retail price of collected samples.

**Figure 3.**
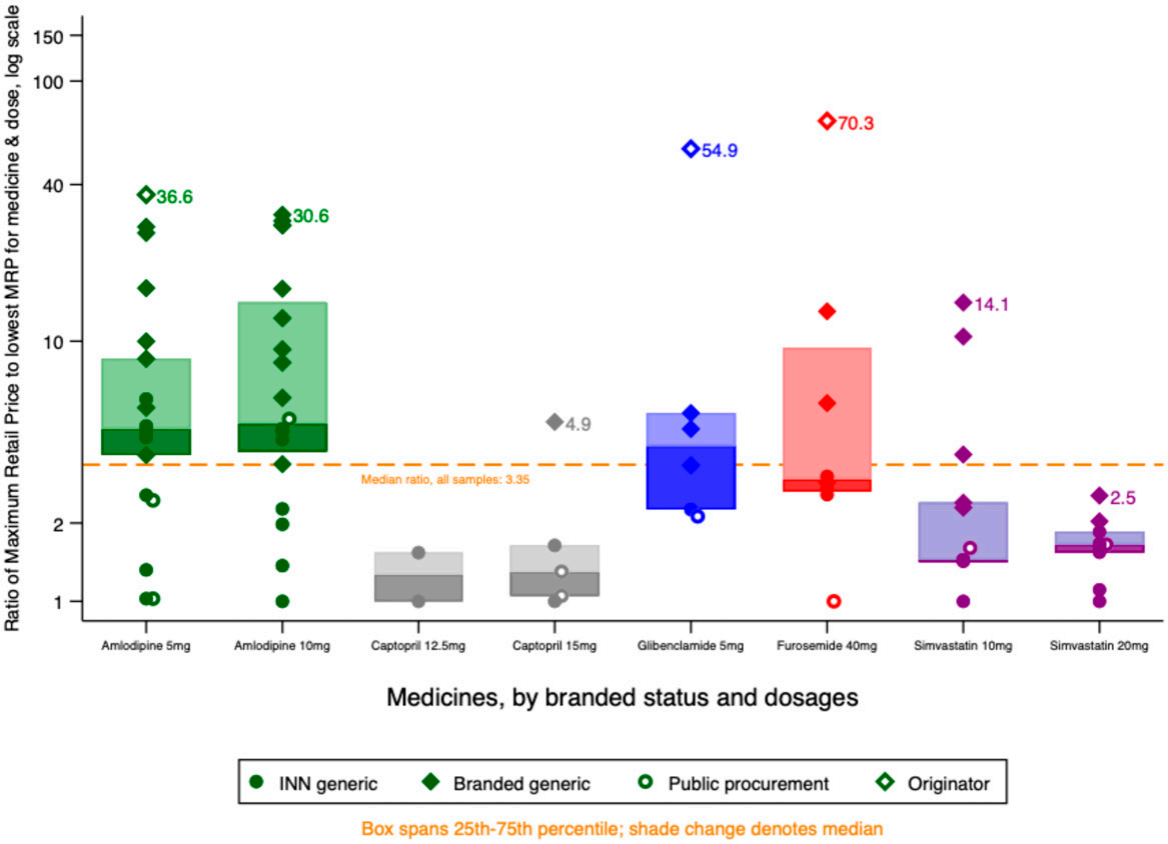
Maximum retail prices by medicine and branded status.

Figure 3 shows the value of the maximum retail price for each of the products in the study relative to the lowest maximum retail price for that API and dose. We use a log scale to preserve detail at the lower price ratios. Manufacturers of originator brands set their highest retail price at 35–75 times that of the cheapest unbranded generic.

Maximum retail prices varied considerably even between unbranded (INN) generics. For furosemide and both doses of amlodipine, we found at least one INN generic version with maximum retail prices higher than the lowest maximum retail price for a branded equivalent.

Some pharmacists said in interviews that brand loyalty was strong even for “unbranded” generic products because patients preferred to stick with manufacturers they knew.

*Lots of patients are, like, if they’re using [an unbranded generic made by] K[imia] F[arma], they don’t want to use Hexpharm. Or if they’re using Hexpharm, they don’t want to use Dexa. So I have to provide all the versions, I have from Dexa, I have from Hexpharm, I have from Kimia Farma. Though really, ah, those three medicines, the content is the same, the composition is the same, the type is the same, but the price can be quite significantly different.*

Pharmacist 1, private pharmacy

Among the study medicines were samples from four holding companies that reflected the range of similar products (same medicine and dosage) they marketed. For three of these companies, we sampled both INN and branded generics, and from a fourth we found an INN generic and two different branded products.

**Figure 4.**
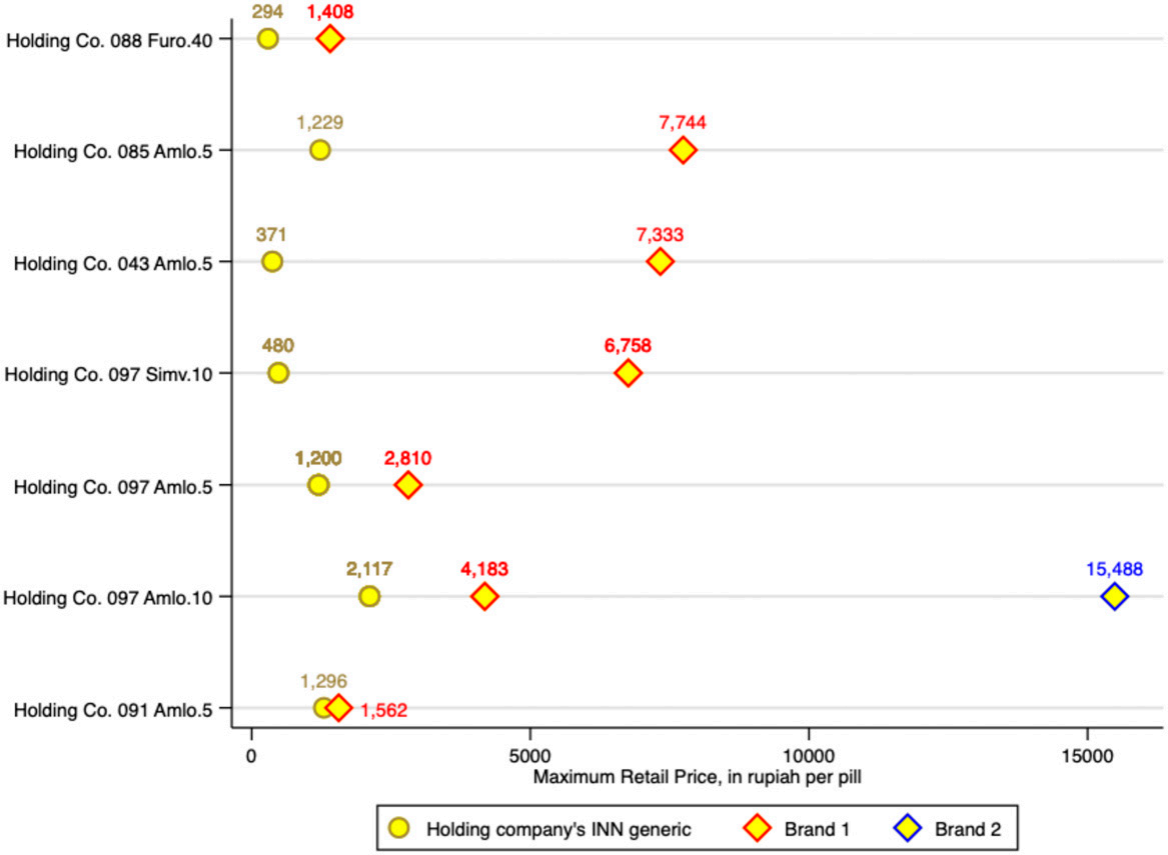
Maximum retail price for branded and unbranded versions of the same product sold by the same company.

Figure 4 shows the varied maximum retail prices set by these companies for their similar products. In this case, we show absolute values rather than ratios to preserve information about variations in base retail prices set for similar products. Whereas Holding Company 44 priced the products similarly (bottom row on Figure 4), the others priced premium products at between twice and 20 times the price of their unbranded products, with a mean ratio of 8.0. At the time of data collection, the exchange rate was US$1 = IDR 14,370.

### Variation in retail prices paid.

**Figure 5.**
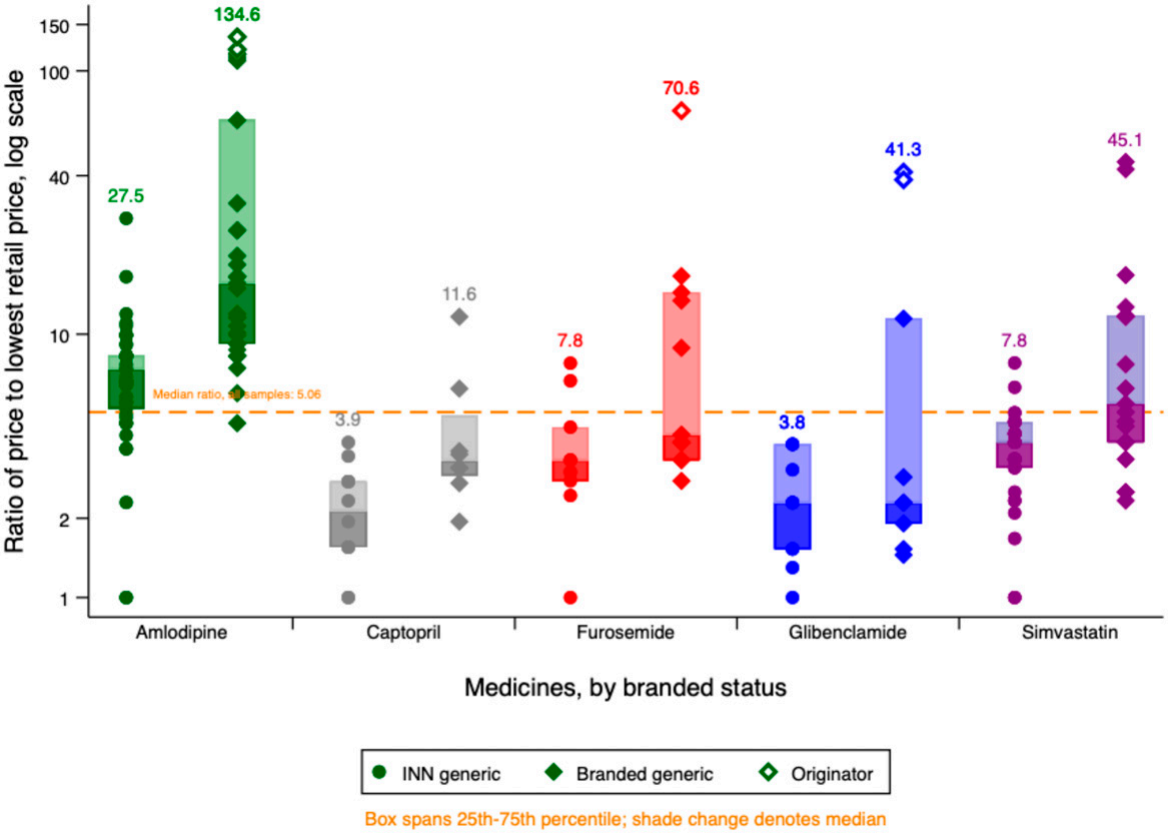
Distribution of relative prices charged by molecule and branded status.

Priced actually paid by patients varied even more than maximum retail prices. Figure 5 shows the ratio of the most expensive to the cheapest price paid for an equivalent product (same API and dose) for all samples that we bought from retail outlets, health providers, or the hospital. The graph differentiates by marker shape between unbranded and branded medicines, showing INN generic medicines on the left and their branded equivalent on the right.

Five brands of amlodipine (comprising seven samples) sold at more than 100-fold the price of the cheapest equivalent product, which was an unbranded generic. For all medicines except captopril, there were brands retailing at 40–71 times the cost of the cheapest medicine. As expected from the distribution of maximum retail prices shown in Figure 3, branded medicines were generally more expensive, However, as Figure 5 shows, the relationship was more varied than maximum retail prices would suggest. For all medicines and dosages, there were unbranded versions (the top circle marker for each medicine) selling for 2–3.7 times the price of the cheapest branded equivalent (the bottom diamond marker). In all cases, however, the cheapest medicine was INN and the most expensive was branded, with originator brands topping the scale where found. Branded medicines traded in a far wider price range than INN medicines.

Supplemental Table 2 summarizes the absolute values underlying the relative values shown in Figure 5, by medicine and dose. Lowest prices by molecule range from 61 rupiah per tablet for amlodipine 5 mg to 239 for simvastatin 20 mg (0.4 US cents and 1.7 cents, respectively); the highest prices range from 1,500 rupiah tablet for captopril 15 mg to 12,650 for amlodipine 10 mg (10.4 cents and 88 cents, respectively).

Retailers reported stocking medicine at a variety of price-points and tailoring their offering to patients based on price signals provided by those patients:

*Like if a patient asks for [the originator brand], well, that’s expensive, so [if I don’t carry it] I have to find a match that’s more or less the same price.*

Pharmacist 1, private pharmacy

*If they’re asking for an unbranded generic, and we don’t have it, what we have a branded generic but it’s also [priced at] 5,000 [rupiah], they’re fine with that… The important thing is that it’s cheap.*

Pharmacist 2, private pharmacy

**Figure 6.**
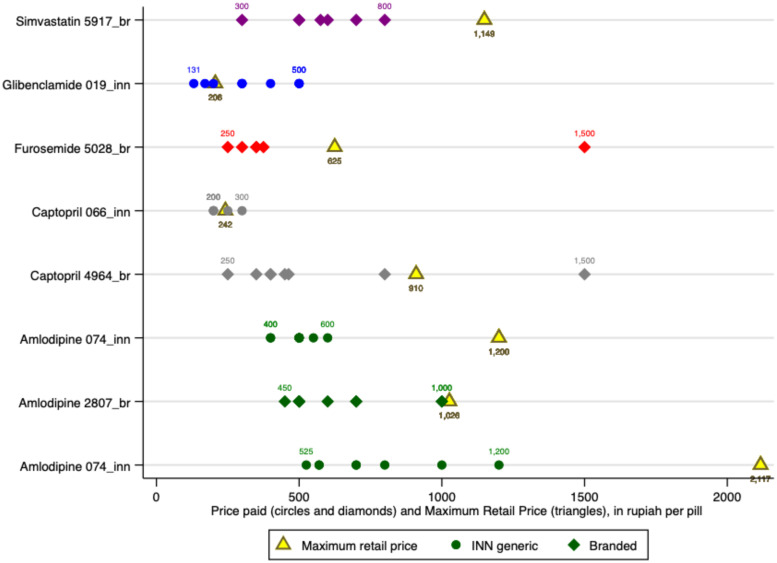
Retail price paid at different outlets and maximum retail price for products of the identical medicine, dosage and brand.

The price variation was not restricted to the difference between brands. Patients in the Malang area were able to find the identical product (same medicine, dose, formulation, and brand or, if unbranded, market authorization holder) at widely varying prices (Figure 6).

In practice, prices for 21% of retail samples exceeded the maximum statutory retail price printed on the packaging; this was significantly more common among medicines sold by doctors and midwives compared with those sold at retail pharmacies/OTC shops or by the hospital (40.7% versus 18.7% and 7.8%, respectively; *P* = 0.016).

**Figure 7.**
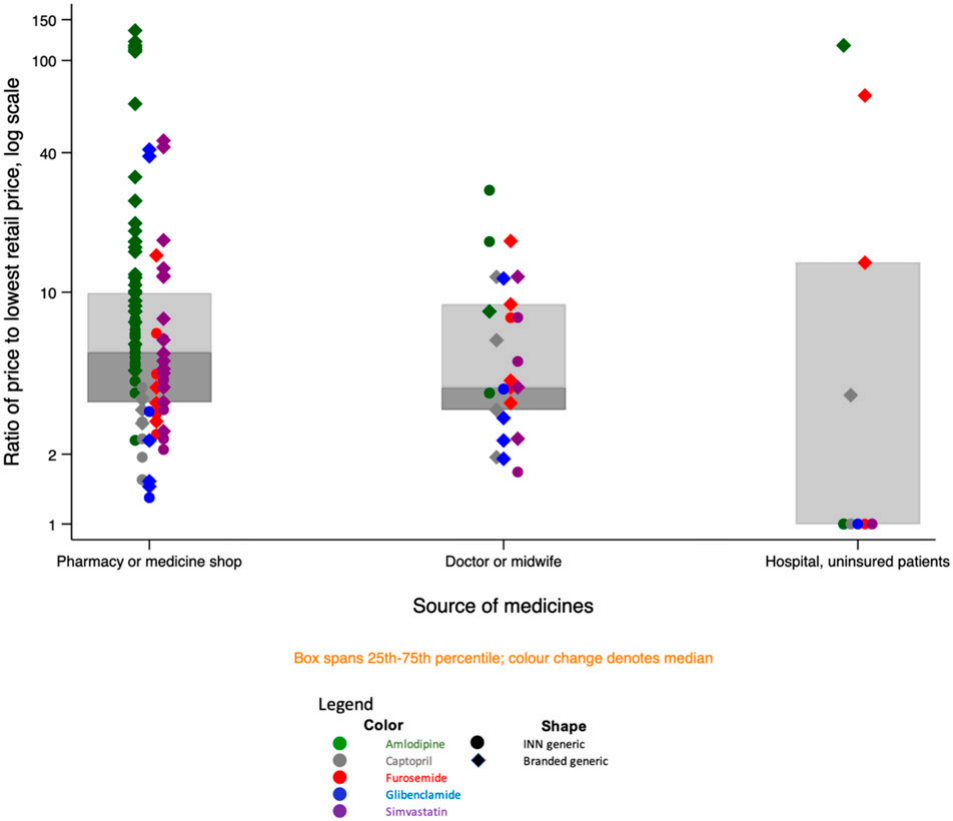
Price variation for similar products, by medicine, branded status, and source.

Figure 7 shows the distribution of relative prices by medicine and source. Uninsured patients buying unbranded generics from the district hospital benefited from the lowest prices for every medicine. However, hospital patients prescribed or choosing branded medicines paid among the highest prices for those products.

Private doctors and midwives charged a wide range of prices; patients buying from these providers paid among the highest prices for unbranded generics.

### Factors influencing choice of medicines on offer.

In interviews, pharmacists and health professionals said that besides wishing to meet patient demand, they decided which products to offer largely on the basis of potential profits or other benefits.

Asked how she chose the distributor or product, one pharmacist replied:

Well it depends. Sometimes I chose another [product or distributor], if there’s a something on offer, maybe a bigger discount. If it’s more profitable then I’d choose that. *laughing*

Pharmacist 1, private pharmacy

In public hospitals, pharmacists have the option of buying medicines for insured patients from the national procurement platform, which at the time of the study was a single-winner system with fixed price products. However, they are not obliged to do so. A hospital pharmacist explained that needed products are in any case not always available on the national platform. In that case, hospital pharmacists order according to the hospital formulary, determined based on doctors’ choices.

*We revise the hospital formulary once every two years, we circulate a list to all the units and doctors: what [brand] do you choose; what do you choose? Then we select three brands, at least the three most common [suggestions].*

Once a brand is on the hospital formulary, administrators will negotiate for volume-based discounts.

The discounts on branded medicines are big, usually, if it’s expensive we can get a big discount…. So we negotiate, to get the maximum discount. For example, like, metamizole, it’s supposed to be in the e-catalog but in fact it’s difficult to find, so we negotiate for A*** (a branded version for metamizole) from the hospital formulary. We negotiate with the producer to give us a maximum discount. At least we try to get the price down close to the e-catalog price, then we can also provide that medicine to the BPJS [publicly-insured] patients.

Pharmacist 2, public hospital

Another interviewee, who previously worked as a senior administrator in a private hospital, described intense lobbying of doctors by pharmaceutical companies aiming to get their products on hospital formularies.

*So in private hospitals … the deal works like this: the (pharmaceutical company) sales staff will give a percent to the hospital and a percent to the doctor. As I remember the hospital got 15%. Of the price of the medicine [paid by the patient]. The doctor got 20%, 10% [downpayment] up front and 10% based on their monthly prescription values. So if I prescribe 10 million worth of [Company X] products, then I get a million in cash that month. That’s for general practitioners; for specialists, the cut is a lot higher.*

Doctor and former private hospital administrator

Individual health care workers also reported being incentivized to provide specific brands.

*[Sales staff] will usually give you some kind of household appliance, like an oven. It’s like: Ma’am, if you take these pills, this many boxes, for a few months, then you’ll get this [reward].*

Private midwife 1

Panel data from two private pharmacies confirmed that, on average, discounts on branded products were greater than on INN generics. As Table 3 shows, the estimated discount averaged 49% for unbranded generics and 62% for branded products. However, because both margins and sale volumes were higher for unbranded generics, these were more profitable for the pharmacies in question.

**Table 3:** Sales data for all versions of study medicines sold at two pharmacies, Malang District

Study medicines	Mean			Total	
	% discount	Margin (rupiah/pill)	Margin (ratio sell: buy price)	Monthly sales, units*	Monthly profits, rupiah*
Branded (*N* = 8)	62.8	82.6	1.3	737	41,878
Unbranded (*N* = 10)	49.0	223.9	2.6	4,563.4	915,769.6
Total	55.2	161.1	2.0	5,300.4	957,647.6

* Average, January–October 2021.

## DISCUSSION

Our study recorded a wide variety of prices for similar cardiovascular and anti-diabetic medicines sold to patients in a largely rural district of East Java but showed that there was no relationship between price and quality. The cheapest medicines all met pharmacopeial specifications, as did equivalent medicines selling at over 100 times the price. This contrasts with other studies, summarized in two systematic reviews largely covering low- and middle-income countries, which reported an average of 15.4% of cardiovascular and 6.8% of diabetes medicines failing at least one quality test.^29^^,^^30^ We speculate that the difference may be in part because all products in our study were produced domestically and are overseen from registration through production and distribution by a single, relatively well-resourced regulator. Indonesia’s national regulator for food and medicine, known as Badan Pengawas Obat dan Makanan (BPOM), is classified as Maturity Level 3 by WHO, the second highest level of performance in the four-level system.^31^ In markets dominated by low-cost imports, it is rarely possible for the national regulator in the consumer country to oversee quality assurance among producers in the way that BPOM does.

The price differentials in the market suggest that both producers and retailers price products at what they believe the market will bear. It begs the question: why do Indonesian patients pay vastly higher prices for premium medicines when they could get products that meet the same pharmacopeial standards at a fraction of the price (or for free, from public services)?

Interviewees provided some evidence that patient choice is influenced by the suggestions of doctors or other healthcare providers. Further, they reported that those actors are sometimes incentivized to dispense or prescribe premium brands by the marketing departments of pharmaceutical companies, in contravention of the code of conduct of GP Farmasi, the Indonesian pharmaceutical trade association.^32^ Qualitative research in other parts of Indonesia suggest that doctors sometimes also have a financial interest in local pharmacies and prescribe to boost profits.^33^

The role of pharmaceutical companies in influencing doctors’ prescribing behavior is evident even in countries with universal health coverage. In France, the competition authority in 2013 fined Sanofi-Aventis over E 40 million for running a smear campaign against generic versions of clopidogrel, which would compete with its Plavix brand.^34^ In other markets where physicians and hospitals have historically profited from the sale of medicines to patients paying out of pocket, such as the United States and China, physicians continue to express distrust of generic medicines (including for cardiovascular disease) despite large-scale studies showing that clinical outcomes do not vary by branding status or price.^4^^–^^6^^,^^35^^–^^38^

In contrast with dynamics in the Indian market reported over a decade ago by Singal et al.,^39^ pharmacists in our study reported receiving bigger discounts from manufacturers of premium products, potentially allowing them to reap significant margins by selling these products. Although the hospital reported negotiating large discounts on branded products, high prices paid by uninsured patients for branded study medicines suggest that discounts are not passed on to consumers. According to Kaplan et al.,^40^ this sort of profit-seeking among those dispensing medicines is common in many settings and stands in the way of successful implementation of policies designed to promote greater use of cost-effective generic medicines.

In our study, however, several pharmacists reported trying to provide products at the varying price points demanded by different patients. We were unable to interview patients directly, which is a major limitation of our study. However, it seems likely that at least some of the demand for premium products in this largely rural area of East Java comes directly from patients because they believe that more money buys better medicine. This would be entirely consistent with the situation reported in other studies, many summarized by Dunne and Dunne.^41^ Poorer patients with limited education are most distrustful of low-cost medicines and especially of those provided free in the public sector.^7^^–^^9^

The WHO asserted in 2017 that 1 in 10 medicines in low- and middle-income countries is substandard or falsified.^42^^,^^43^ However, the few medicine quality surveys that report a relationship between price and quality do not suggest that cheaper or INN generic medicines are any more likely to be poor quality than more expensive or branded medicines. Testing noncommunicable disease medicines in Cambodia between 2011 and 2013, Rahman et al.^44^ found that noncompliant samples of glibenclamide were twice as expensive, on average, as those that passed testing, whereas for amlodipine there was no relationship. A small study comparing premium (“branded”) and nonpremium (“branded-generic”) pairs of medicines produced by the same manufacturer in the Indian market found that all met quality standards.^39^ Among 92 samples of 12 essential medicines collected in Togo, samples that were relatively cheaper compared with an international standard were not significantly more likely to fail than relatively expensive samples.^45^

In our study, one doctor, practicing some years ago, described witnessing treatment failure in patients using low-cost unbranded products, which resolved after switching them to branded products. After the introduction of the national health insurance scheme JKN, and with it free medicines in public facilities, the Indonesian press regularly reported concerns about the quality of those medicines.^17^^,^^46^ Although in our study a government pharmacist reported a steady reduction in quality problems in recent years, it is possible that perceptions of ineffective or otherwise poor quality cheap medicines rooted in experience have survived, even as actual product quality has improved through investment in production processes and better regulation. Studies in the United States and New Zealand have also demonstrated that the perception of quality equated with price can have a strong placebo effect, even in the absence of active ingredients.^47^^–^^49^

Many of the prices in the Indonesian market, including a majority of those in the public procurement system, are now well below the last iteration of the international reference price, adjusted for inflation.^50^ Among 83 unique versions of four cardiovascular and one anti-diabetic medicine in Indonesia, comprising a total of 204 samples collected to reflect the likelihood of patient exposure to particular products in a district in East Java, we found no factual basis for any lingering perception that cheap medicines are of poor quality. In a large, economically and socially diverse market such as Indonesia’s, it is possible that the quality of low-cost items is protected in part through cross-subsidization on the part of manufacturers. Regulatory data indicate that it is extremely common for Indonesian companies to register more than one version of the same product—most commonly a branded and an unbranded version from a single market authorization holder. Under Indonesian regulations, these must be identical in composition. However, market authorization holders are free to set their own maximum retail prices for branded products. The wide variation in maximum retail prices suggests companies are engaging in market segmentation, providing products at different price points to increase their overall share of the market. In the “paired” products in our study area (INN and branded versions of the same product from the same holding company), companies set prices up to 20 times higher from their branded products compared with the unbranded generic equivalent.

From monthly data recorded by two pharmacies, we estimated the discounts at which pharmacies acquire medicines and found that they were greatest for branded medicines. However, the margin percentage on unbranded generics was on average double that on branded medicines; coupled with higher sales volumes for INN generics, this made these unbranded products more profitable. This observation is based on limited data; we could not verify the wholesale prices at which most pharmacies acquired medicines. However, price variation between pharmacies for the identical product suggests that pharmacists also adapt sales prices to achieve maximum profits, for example by charging lower margins on higher-priced products or those acquired at a greater discount while pushing up margins on lower-priced, higher-volume brands.

In practice, prices charged to consumers were lower than the maximum retail price four-fifths of the time. This suggests two things: 1) that manufacturers set maximum retail prices (MRPs) at aspirational levels that allow retailers plenty of room to make a profit and 2) that the “transparency” afforded by printed MRPs protects consumers from additional price gouging in the regulated supply chain, where the medicine regulator audits sales invoices. In our study, those charging over the MRP were often unregulated dispensers (doctors, midwives, and medicine shops), who may buy their own medicine supply from retail pharmacies and who are not subject to oversight from regulators or corporate management boards, as is the case in many health facilities and chain pharmacies.

Some politicians, including a former Indonesian health minister, claim that high-priced medicines are an enduring burden for Indonesian patients.^51^ Concerns have also been raised that the “single-winner per province” system has caused shortages and impeded access to medicines for insured patients.^13^^,^^52^ This is especially likely to be true in more remote areas of the country and in districts that have a history of late payment through the public procurement system.^33^ However, for the five study medicines, among the most commonly consumed in Indonesia, we found that procurement policies in force at the time of the study, including consolidated tendering at the national level and price transparency in the public procurement system, have been broadly successful in making quality-assured products widely available to patients free at point of care in a largely rural area in one of the nation’s most densely populated regions. This has been achieved at a cost that does not excessively burden the public insurer’s budget.

These largely successful policies were revised, effective January 2023, in an apparent effort to reduce unquantified stockouts.^53^^,^^54^ Consolidated tenders have been dropped. Each health facility is now free to negotiate with manufacturers, and prices paid will no longer be visible to the public. These changes run counter to WHO recommendations for medicine pricing policy.^55^ Fragmentation of demand will reduce negotiating power and could lead to higher prices, and the loss of transparency may encourage reversion to the pre–e-catalogue kickback system described in this paper. However, the real effects of the changes on access, price, and quality of medicines in the public health system in Indonesia remain to be seen.

For those unwilling or unable to access free medicines from public services, very low cost, quality-assured products are widely available in pharmacies. The simultaneous existence of high-priced alternatives for those willing and able to pay for the illusion of better quality is thus not problematic. Indeed, if producers sell expensive branded products to wealthier Indonesians and use some of the profits thus generated to support the sale of very low-cost equivalent INN products to the public health system and to poorer patients, the price variation in the market may benefit health for all Indonesians.

We note that our budget only allowed us to test for identity, percentage of labeled assay, and dissolution. It is possible that the tested medicines were not uniform in content, contained impurities, or suffered from other defects and that these defects were more common among lower-priced medicines. In addition, the study was carried out in a largely rural area of the most developed and densely populated island, Java; it is possible that lower-priced medicines degrade more rapidly than those sold at higher prices and are thus more likely to sink below the quality threshold as they get transported thousands of kilometers to the country’s outer islands.

Overall, however, we conclude that the millions of Indonesian patients who take amlodipine, captopril, furosemide, glibenclamide, or simvastatin daily can be broadly confident that their medicines are of acceptable quality, regardless of the price they paid.

## Financial Disclosure

This research was funded by the National Health and Medical Research Council (NHMRC) of Australia under grant number NHMRC APP1149987.

## Supplemental Materials


Supplemental materials
