# Supplementary material for: Variation in Price of Cardiovascular and Diabetes Medicine in Indonesia, and Relationship with Quality: A Mixed Methods Study in East Java
Source: Am J Trop Med Hyg. 2023 May 9;108(6):1287–99. doi: 10.4269/ajtmh.22-0692 (PMC10540131; doi:10.4269/ajtmh.22-0692)
Supplement: Supplementary file 1 [file tpmd220692.SD1.pdf]

Supplementary Table 1: Profile of In-depth Interview contributors

Contributor	Sector	Number of individuals interviewed
**District Warehouse**	Public	2
**Primary health centre (*Puskesmas*)**	Public	4
**Community health outpost**	Public	2
**District hospital**	Public	1
**Local medicine distributor**	Private	1
**Independent pharmacy**	Private	3
**National chain pharmacy**	Private	2

Supplementary Table 2: Details of medicine prices, by medicine and dosage

	Prices in Indonesian Rupiah					Prices in US$					
	Lowest	25th percentile	Median	75th percentile	Highest	Lowest	25th percentile	Median	75th percentile	Highest	# samples
Branded											
Amlodipine 5mg	450	500	1000	1500	8145	0.0313	0.0348	0.0696	0.1044	0.5668	19
Amlodipine 10mg	500	1000	1664	12000	12650	0.0348	0.0696	0.1158	0.8351	0.8803	16
Captopril 15mg	250	375	425	632	1500	0.0174	0.0261	0.0296	0.0439	0.1044	8
Glibenclamide 5m	190	250	300	1500	5400	0.0132	0.0174	0.0209	0.1044	0.3758	9
Furosemide 40mg	250	300	375	1300	6369	0.0174	0.0209	0.0261	0.0905	0.4432	11
Simvastatin 10mg	300	575	750	1513	5800	0.0209	0.0400	0.0522	0.1053	0.4036	14
Simvastatin 20mg	600	600	800	1833	4000	0.0418	0.0418	0.0557	0.1276	0.2784	5
Unbranded											
Amlodipine 5mg	61	400	500	550	1667	0.0042	0.0278	0.0348	0.0383	0.1160	29
Amlodipine 10mg	109	500	600	750	1300	0.0076	0.0348	0.0418	0.0522	0.0905	21
Captopril 12.5mg	73	200	200	200	250	0.0051	0.0139	0.0139	0.0139	0.0174	5
Captopril 15mg	129	200	200	300	500	0.0090	0.0139	0.0139	0.0209	0.0348	7
Glibenclamide 5m	131	200	300	500	500	0.0091	0.0139	0.0209	0.0348	0.0348	11
Furosemide 40mg	90	250	300	400	700	0.0063	0.0174	0.0209	0.0278	0.0487	9
Simvastatin 10mg	129	400	500	600	1000	0.0089	0.0278	0.0348	0.0418	0.0696	14
Simvastatin 20mg	239	800	1000	1003	1500	0.0166	0.0557	0.0696	0.0698	0.1044	17
